# Supplementary material for: Learning to think critically about health using digital technology in Ugandan lower secondary schools: A contextual analysis
Source: PLoS One. 2022 Feb 2;17(2):e0260367. doi: 10.1371/journal.pone.0260367 (PMC8809610; doi:10.1371/journal.pone.0260367)
Supplement: S2 File — (DOCX) [file pone.0260367.s003.docx]

Links to the learning resources under development.

· Whole site: [https://choice-teachers.epistemonikos.org](https://choice-teachers.epistemonikos.org/)

· Student computer-based lessons (no menu or access to rest of site)

<https://choice-teachers.epistemonikos.org/students>
