# Supplementary material for: Learning to think critically about health using digital technology in Ugandan lower secondary schools: A contextual analysis
Source: PLoS One. 2022 Feb 2;17(2):e0260367. doi: 10.1371/journal.pone.0260367 (PMC8809610; doi:10.1371/journal.pone.0260367)
Supplement: S3 File — (DOCX) [file pone.0260367.s004.docx]

ICT School Survey conducted via Telephone

Take note of extra information that may be important

Good morning/afternoon Mr./Mrs………………. .I am ……………..., calling from Makerere University College of Health Sciences. We got your contacts from the Minitsry of Education and Sports as the head teacher/propriator of ………………. We are coducting an ICT school survey as part of the Informed Health Choices Project and would like to speak to you for about 10-15 minutes. The Informed Health Choices Project aims to empower students with ability to assess the trustworthiness of health calims (things people say about health) by developing digital learning materials.

Consent: Is it OK, if we ask a few questions about digital resources now? (Yes, No)

1. What type(s) of ICT devices does your school have? (Check all that apply, DO NOT READ OUT LOUD)

*(e.g. computer*)

| Equipment | Available?  (Yes/No) | Functional  (Yes/No) | Used while teaching (Yes/No) |
| --- | --- | --- | --- |
| Computers |  |  |  |
| Projectors |  |  |  |
| If a projector malfunctions, about how long does it take to have it repaired? |  | | |
| Tablets |  |  |  |
| Electronic boards |  |  |  |
| Public Address System |  |  |  |
| Television with video input devices (DVD, Flask drive) |  |  |  |
| Other, specify |  |  |  |
| No ICT devices |  |  |  |
| Does the school have internet? |  |  |  |

1. Does the school have an IT specialist/Technician/Teacher? (Support structures for use of ICT in the school) (Yes/No)
2. Ratio of computers to students (i.e 1:2)? __________________
3. Non ICT teacher’s perception towards ICT

| Learning resources (For non-ICT teachers) | |
| --- | --- |
| Do non- ICT teachers deliver lessons using ICT/digital devices? *(Yes/No)* |  |
| How common do teachers use smartphones to access and teach content during a lesson? *(1-Very common, 2-Quite common, 3-rarely, 4-Not at all)* |  |
| How confident are the non-ICT teachers at delivering lessons using ICT devices?  *(1-Very confident, 2-Confident, 3-Neutral, 4-Not confident, 5-Not confident at all)* |  |

1. When do students/teachers access and use the computers for learning?

| 5a. | Tick as appropriate | |
| --- | --- | --- |
|  | students | teachers |
| Only during classes |  |  |
| During classes and outside study time |  |  |
| Only outside study time |  |  |

1. Power/Electricity and Back-up systems: Does the school have power/electricity source?

1-No, 2-Yes, electricity, 3-Yes, electricity & stand by generator, 4-Yes, electricity & solar sytem

5-Yes, electricity & stand by generator & solar sytem

1. How are learning resources shared and accessed to students?

| Distribution of learning resources (Senior 1, 2 & 3) | Yes/No  (Choose all that apply or specify) |
| --- | --- |
| **Shared by teachers** |  |
| Printed paper notes |  |
| Dictation (reading to students) |  |
| Writing on boards |  |
| Central server |  |
| School website |  |
| Social media sites (WhatsApp/Facebook/YouTube) |  |
| Text books |  |
| Zoom |  |
| Skype |  |
| Other, specify. |  |
| **Accessed by students** |  |
| Downloaded directly to the computers for students to access |  |
| Downloaded to a local network of computers |  |
| WhatsApp |  |
| Other, specify. |  |
| Comments | __________________________ |

Comments: _______________________________________________

**Thank participant for their time.**
