# Supplementary material for: Learning to think critically about health using digital technology in Ugandan lower secondary schools: A contextual analysis
Source: PLoS One. 2022 Feb 2;17(2):e0260367. doi: 10.1371/journal.pone.0260367 (PMC8809610; doi:10.1371/journal.pone.0260367)
Supplement: Attachment — Submitted filename: Response to Reviewers.docx [file pone.0260367.s005.docx]

Dear Academic Editor,

Re: Response the comments on our manuscript; PONE-D-21-14333
Learning to think critically about health using digital technology in Ugandan lower secondary schools: a contextual analysis.
PLOS ONE

We wish to thank you for the helpful comments. We have carefully reviewed and addressed each comment as detailed below;

**Comments from the academic editor**

1. Please ensure that your manuscript meets PLOS ONE's style requirements, including those for file naming. The PLOS ONE style templates can be found at

<https://journals.plos.org/plosone/s/file?id=wjVg/PLOSOne_formatting_sample_main_body.pdf> and

<https://journals.plos.org/plosone/s/file?id=ba62/PLOSOne_formatting_sample_title_authors_affiliations.pdf>

***Response: We have checked the PLOS ONE style requirements from the guidelines above and edited our paper to ensure that we adhere to them.***

2. Please include your tables as part of your main manuscript and remove the individual files. Please note that supplementary tables (should remain/ be uploaded) as separate "supporting information" files

***Response: We have included the tables as part our main manuscript and removed the individual files as guided.***
3. We note that the grant information you provided in the ‘Funding Information’ and ‘Financial Disclosure’ sections do not match.

***Response: We have corrected this and now the grant information in both sections matches.***


**Reviewers' comments:**

Reviewer #1:

1. A wider international audience may need one sentence of context about the song "Alone and Frightened" and/or a link to information.

***Response: We have added a reference on page 23 (see line 493 in the revised manuscript track changes version). We think that the reference would provide more details about the song than a sentence.***

2. The description of the availability of electricity isn't sufficiently clear. It seems that overall 71% of schools have mains electricity (that is predominantly hydroelectric power in Uganda) and the remainder have an electricity generator or solar power generated locally.

***Response: We have re-written this paragraph to clarify that only 71% of the schools had hydroelectric power which is the most common source of power in schools and that 29% did not have access to any form of electricity on page 21 (see line 456-460 in the revised manuscript track changes version).***

3. There is insufficient comparison with the results of a recently published research work that has several authors in common and on a very similar topic in reference 22 (Mugisha M, Uwitonze AM, Chesire F, Senyonga R, Oxman M, Nsangi A, et al. Teaching critical thinking about health using digital technology in lower secondary schools in Rwanda: A qualitative context analysis. PLoS One. 2021;16(3).).

***Response: We have improved the comparison by adding a paragraph that enriches the comparison as well as referencing the areas where similar findings were found on page 32 (see line 548 in the revised manuscript track changes version).***

Reviewer #2:

This paper addresses an extremely interesting topic. Critical thinking skills about health, or Critical Health Literacy, could be taught, but there are few interventions existing and it is necessary to investigate their opportunity and feasibility. The authors have conducted a thorough contextual analysis, but I believe the manuscript in its present form it is suitable for publication and does not do justice to the work. Below my main concerns:
• The authors list a number of research questions in the introduction and describe a number of studies in the methods section. However, it is not clear which study helps answering which research question. I believe this should be specified.

***Response: We have edited the first paragraph in the methods section on page 6 (see line 128 and 136 in the revised manuscript track changes version) to clarify which objectives are addressed by the different methods that we used.***

• In the methods section some information is missing throughout.
o Document analysis: When listing the documents, it is not clear how they have been selected and why. In addition, information is missing on the process of analysis (inductive?) and on how table 1 has been derived. It is not clear to me what “two-degree holders” means, it not specific of expertise of the reviewers.

***Response: We have added a sentence that justifies why and how we selected the listed documents. Documents relevant to critical thinking about health and ICT in education were selected from the website searches and office visits (see line 148 to 150 on page 7 in the revised manuscript track changes version). We have also specified that we conducted deductive analysis of the documents (see line 161 in the revised manuscript track changes version). We have also rewritten the “two-degree holders” to two research assistants to demonstrate the expertise of the reviewers on page 7 (see line 168 in the revised manuscript track changes version) all on page 7***

o Workshops: the description of the workshops is not specific about their structure. How were participants informed beforehand? How was NGT implemented? Also, wen mentioning prototypes in workshop 2, more details would be appreciated.

***Response: We have added a sentence about how participants were informed beforehand on page 8 (see line 179 and 181 in the revised manuscript track changes version). The NGT was implemented as indicated in the five bullet points on page 8 (see line 187 to 192 in the revised manuscript track changes version). We have provided more details about the prototypes in workshop 2 for on page 9 (see line 202 to 204 in the revised manuscript track changes version).***

o Qualitative interviews: when explaining data collection the authors indicate, “we excluded questions that were already covered satisfactorily in the preceding interviews and modified the guide by adding questions that needed more exploration”. Could you be more clear about this? Was analysis undergoing in parallel? Do you mean that data saturation was achieved? More information about the analysis is needed as well.

***Response: We have clarified that the analysis was conducted as data were collected and that questions were removed when saturation was achieved on page 9 (see line 224 to 225 in the revised manuscript track changes version). Data saturation was assessed by checking whether similar responses were obtained within and across respondent categories as detailed in the description of the analysis on page 11 (see line 275 to 277 in the revised manuscript track changes version).***

o School survey: could you please provide more information about the questionnaire?

***Response: We have added a sentence at the end of the school survey paragraph under methods that shows the sub-sections that we included in the questionnaire when conducting the school survey among head teachers on page 11 (see line 264 and 265 in the revised manuscript track changes version).***

• The results section presents results from the different studies, but is not evident which study contributed to what. It would be good, in my opinion, to clarify this both in the introduction to the results section and in the other subchapters.

***Response: We have addressed this in the first paragraph of the Methods section, as indicated above in response to this reviewer’s first comment. In the results section, we have indicated which methods have contributed to each section: on page 15 (see line 312 to 314 in the revised manuscript track changes version).***

***Page 15 line 328, page 17 line 383, page 18 line 410. In addition, the sources for each key finding are specified in Table 6.***

• I am getting lost in the second part of the discussion (section 4 and 5) as I do not see the direct link with the purpose of the study and the results presented. Maybe it would be worth it to elaborate this content further.

***Response: We have added a sentence at the beginning of each of those paragraphs clarifying how they related to the results on page 31 (see lines 551 to 552 and 564 to 566 and in the revised manuscript track changes version)***
